# Supplementary material for: Modelling Skylarks (Alauda arvensis) to Predict Impacts of Changes in Land Management and Policy: Development and Testing of an Agent-Based Model
Source: PLoS One. 2013 Jun 6;8(6):e65803. doi: 10.1371/journal.pone.0065803 (PMC3675089; doi:10.1371/journal.pone.0065803)
Supplement: Supporting Information S4 — The skylark ODdox as a zipped archive. (ZIP) [file pone.0065803.s004.zip › Skylark_ODdox/class_movement_map16.html]

ALMaSS Skylark ODdox: MovementMap16 Class Reference


ALMaSS Skylark ODdox
 2.0


- Main Page
- Related Pages
- Classes
- Files

- Class List
- Class Index
- Class Hierarchy
- Class Members

Public Member Functions |
Public Attributes |
Protected Member Functions |
Protected Attributes

MovementMap16 Class Reference

Movement maps are used for rapid computing of animal movement.
More...

`#include <MovementMap.h>`

List of all members.

|  |  |
| --- | --- |
| Public Member Functions | |
| void | ClearMapValue (unsigned x, unsigned y) |
| int | GetMapValue (unsigned x, unsigned y) |
|  | MovementMap16 (Landscape \*L) |
| void | SetMapValue (unsigned x, unsigned y, unsigned value) |
|  | ~MovementMap16 () |

|  |  |
| --- | --- |
| Public Attributes | |
| uint32 \* | m\_TheMap |
| uint32 | maxx |
| uint32 | maxy |

|  |  |
| --- | --- |
| Protected Member Functions | |
| void | Init () |

|  |  |
| --- | --- |
| Protected Attributes | |
| Landscape \* | m\_ALandscape |

---

## Detailed Description

Movement maps are used for rapid computing of animal movement.

---

## Constructor & Destructor Documentation

|  |  |  |  |  |  |
| --- | --- | --- | --- | --- | --- |
| MovementMap16::MovementMap16 | ( | Landscape \* | *L* | ) |  |

References Init(), m\_ALandscape, m\_TheMap, maxx, maxy, Landscape::SupplySimAreaHeight(), and Landscape::SupplySimAreaWidth().

{

m\_ALandscape = L;

// must make sure that we have whole words

maxx=(m\_ALandscape->SupplySimAreaWidth()+1)/2;

maxy=m\_ALandscape->SupplySimAreaHeight();

m\_TheMap = new uint32[ maxx\*maxy ];

Init();

}

|  |  |  |  |  |
| --- | --- | --- | --- | --- |
| MovementMap16::~MovementMap16 | ( |  | ) |  |

References m\_TheMap.

{

delete m\_TheMap;

}

---

## Member Function Documentation

|  |  |  |  |
| --- | --- | --- | --- |
| void MovementMap16::ClearMapValue | ( | unsigned | *x*, |
|  |  | unsigned | *y* |
|  | ) |  |  |

References m\_TheMap, and maxx.

{

uint32 theWord=x & 1; // is the last bit set?

theWord=theWord<<4; // Multiply by 16

// divide by 2

uint32 index= (x>>1)+(y\*maxx); // y and x/2

m\_TheMap[index] &= ~(0xFFFF<< theWord);

}

|  |  |  |  |
| --- | --- | --- | --- |
| int MovementMap16::GetMapValue | ( | unsigned | *x*, |
|  |  | unsigned | *y* |
|  | ) |  |  |

References m\_TheMap, and maxx.

{

uint32 theWord=x & 1;

// Multiply by 16

theWord=theWord<<4;

// divide by 16

uint32 index= (x>>1)+(maxx\*y);

return ((m\_TheMap[index] >> theWord) & 0xFFFF); //

}

|  |  |  |  |  |
| --- | --- | --- | --- | --- |
| void MovementMap16::Init | ( |  | ) |  |

protected

References m\_ALandscape, SetMapValue(), Landscape::SupplySimAreaHeight(), and Landscape::SupplySimAreaWidth().

Referenced by MovementMap16().

{

for (int y=0; y<m\_ALandscape->SupplySimAreaHeight(); y++)

{

for (int x=0; x<m\_ALandscape->SupplySimAreaWidth(); x++)

{

SetMapValue(x,y,0);

}

}

}

|  |  |  |  |
| --- | --- | --- | --- |
| void MovementMap16::SetMapValue | ( | unsigned | *x*, |
|  |  | unsigned | *y*, |
|  |  | unsigned | *value* |
|  | ) |  |  |

References m\_TheMap, and maxx.

Referenced by Init().

{

uint32 theWord= x & 1; // is the last bit set?

// Multiply by 16o

theWord=theWord<<4;

// divide by 16

// Calculate index:

uint32 index= (x>>1)+(y\*maxx);

value &= 0xFF;

uint32 NewVal = m\_TheMap[index] & ~( 0xFFFF << theWord );

m\_TheMap[index] = NewVal | (value << theWord);

}

---

## Member Data Documentation

Landscape\* MovementMap16::m\_ALandscape

protected

Referenced by Init(), and MovementMap16().

uint32\* MovementMap16::m\_TheMap

Referenced by ClearMapValue(), GetMapValue(), MovementMap16(), SetMapValue(), and ~MovementMap16().

uint32 MovementMap16::maxx

Referenced by ClearMapValue(), GetMapValue(), MovementMap16(), and SetMapValue().

uint32 MovementMap16::maxy

Referenced by MovementMap16().

---

The documentation for this class was generated from the following files:

- MovementMap.h
- MovementMap.cpp


- MovementMap16
- Generated on Thu Jan 10 2013 13:15:36 for ALMaSS Skylark ODdox by
   1.8.1.1
